# Supplementary material for: Content-rich biological network constructed by mining PubMed abstracts
Source: BMC Bioinformatics. 2004 Oct 8;5:147. doi: 10.1186/1471-2105-5-147 (PMC528731; doi:10.1186/1471-2105-5-147)
Supplement: Additional File 2 — The original results of the above study (non-essential files are deleted to keep the file size under the limit set by BMC bioinformatics). [file 1471-2105-5-147-S2.bz2 › chilibotAdditionalFile2/dip30/231ID7931156E750/html/UL52_UL8.html]

 


 **UL52** and **UL8** 
  
Found 43 abstracts in PubMed, retrieved 30.  
 

 What does Google say? 
 PDF only 
| .edu only 

---

**Interactive relationship** (e.g. stimulation, inhibition, etc)

**Neutral relationship**- Two hybrid analysis of the interaction between the  **UL52**  and  **UL8**  subunits of the herpes simplex virus type 1 helicase primase.  Ref: 10501495 J Gen Virol, 1999
- In contrast, the  **UL52**   **UL8**  interaction gave a strong signal in the two hybrid system.  Ref: 10501495 J Gen Virol, 1999
- The interactions of the  **UL52**  protein with the  **UL8**  and UL5 proteins were analysed by using the yeast two hybrid system.  Ref: 10501495 J Gen Virol, 1999
- Inhibition of herpes simplex virus replication by a 2 amino thiazole via interactions with the helicase component of the UL5  **UL8**   **UL52**  complex.  Ref: 9696789 J Virol, 1998
- The herpes simplex virus type 1  **UL8**  protein influences the intracellular localization of the  **UL52**  but not the ICP8 or POL replication proteins in virus infected cells.  Ref: 8811024 J Gen Virol, 1996
- Reconstitution of lagging strand synthesis in the presence of UL5  **UL52**  was achieved by the addition of partially purified  **UL8** .  Ref: 1321275 J Virol, 1992
- Experiments performed using extracts from doubly infected cells indicated that  **UL8**  could interact separately with both the UL5 and  **UL52**  proteins.  Ref: 7931156 J Gen Virol, 1994
- Deletion analysis showed that a 548 amino acid fragment of  **UL52**  amino acids 366 914 retains the ability to interact with  **UL8** .  Ref: 10501495 J Gen Virol, 1999
- The ability of these three mutant UL5 proteins to form specific interactions with other members of the helicase primase complex,  **UL8**  and  **UL52** , indicates that the functional domains required for replication activity of UL5 are separable from domains responsible for protein protein interactions.  Ref: 1309257 J Virol, 1992
- Deletion mutants of the herpes simplex virus type 1  **UL8**  protein effect on DNA synthesis and ability to interact with and influence the intracellular localization of the UL5 and  **UL52**  proteins.  Ref: 9344911 Virology, 1997
- MAb 0811 was shown to recognize the  **UL8**  protein in both Western blots and immunoprecipitation assays and to co precipitate the other two proteins in the complex from insect cells triply infected with recombinants expressing the UL5,  **UL8**  and  **UL52**  polypeptides.  Ref: 7931156 J Gen Virol, 1994
- The  **UL8**  component of the herpes simplex virus helicase primase complex stimulates primer synthesis by a subassembly of the UL5 and  **UL52**  components.  Ref: 8106478 J Biol Chem, 1994

**Non-interactive relationship** (e.g. studied together, co-existance, homology, etc.)

- The DNA helicase primase complex of HSV 1 consists of three sub units with molecular weights of 1 0 00 and 7 00, encoded by genes  **UL52** , UL5 and  **UL8** , respectively.  Ref: 9421876 Virus Genes, 1997
- Herpes simplex virus type 1 encodes a helicase primase complex composed of the products of the UL5,  **UL52** , and  **UL8**  genes.  Ref: 8662872 J Biol Chem, 1996
- Since this pattern can form in cells infected with mutants which are defective in UL5,  **UL8** , UL9, or  **UL52**  in the presence of polymerase inhibitors C.  Ref: 9151871 J Virol, 1997
- All three subunits of this complex UL5,  **UL8** , and  **UL52**  are required for viral DNA replication in all cell types.  Ref: 9445019 J Virol, 1998
- These studies reveal that in the absence of ICP27, expression of the UL5,  **UL8** ,  **UL52** , UL9, UL42, and UL30 genes is significantly reduced at the level of mRNA accumulation.  Ref: 8627723 J Virol, 1996
- The herpes simplex virus type 1 HSV UL5,  **UL8** , and  **UL52**  proteins form a helicase primase complex in infected cells.  Ref: 8106478 J Biol Chem, 1994
- T157602 inhibited reversibly the helicase activity of the HSV UL5  **UL8**   **UL52**  UL5 8 52 helicase primase complex with an IC50 concentration of compound that yields 50% inhibition of 5 microM.  Ref: 9696789 J Virol, 1998
- We constructed recombinant viruses based on the herpes simplex virus type 1 mutant tsK which individually were able to express the products of four viral DNA replication genes UL5,  **UL8** , UL9 and  **UL52**  in the absence of any of the other proteins required for viral DNA synthesis.  Ref: 1312116 J Gen Virol, 1992
- The herpes simplex virus type 1 UL5,  **UL8** , and  **UL52**  genes encode an essential heterotrimeric DNA helicase primase that is responsible for concomitant DNA unwinding and primer synthesis at the viral DNA replication fork.  Ref: 9593724 J Biol Chem, 1998
- The herpes simplex virus HSV type 1 helicase primase is a three protein complex, consisting of a 1 1 1 association of UL5,  **UL8** , and  **UL52**  gene products J.J.  Ref: 1321275 J Virol, 1992
- The assembly of complete HSV replication compartments and incorporation of BrdU were both abolished by treatment with phosphonoacetic acid and by omission of any one of the seven viral replication proteins, UL5,  **UL8** , UL9, UL42,  **UL52** , SSB, and Pol, that are essential for viral DNA replication.  Ref: 9060678 J Virol, 1997
- The ability of the  **UL8**  mutants to facilitate efficient nuclear localization of  **UL52**  in the presence of coexpressed UL5 was examined by immunofluorescence.  Ref: 9344911 Virology, 1997
- In BHK cells infected with ambUL8, a mutant with an amber termination codon within the coding region of gene  **UL8** , the  **UL52**  protein did not enter the nucleus, although ICP8 and POL entered the nucleus in a normal fashion.  Ref: 8811024 J Gen Virol, 1996
- We also found that the primers made by the subcomplex are equal in length to those synthesized by the UL5  **UL8**   **UL52**  complex.  Ref: 1321275 J Virol, 1992
- The Herpes simplex virus type 1 primosome consists of three subunits that are the products of the UL5,  **UL8** , and  **UL52**  genes.  Ref: 9278436 J Biol Chem, 1997
- We observed that four replication proteins, UL5,  **UL8**   **UL52** , and UL9, are necessary for the localization of ICP8 UL29 to prereplicative sites natural infection conditions.  Ref: 8627698 J Virol, 1996
- Herpes simplex virus type 1 expresses a heterotrimeric helicase primase, the subunits of which are encoded by the viral UL5,  **UL8**  and  **UL52**  genes.  Ref: 10501495 J Gen Virol, 1999
- On the basis of these results, we present a model for prereplicative site formation in infected cells in which the helicase primase components UL5,  **UL8** , and  **UL52** , the origin binding protein UL9, and the viral single stranded DNA binding protein ICP8 assemble together to initiate the process.  Ref: 8627698 J Virol, 1996
- The results demonstrated that all three components of the viral helicase primase complex UL5,  **UL8**  and  **UL52**  proteins must be co expressed to allow their efficient localization to the nucleus.  Ref: 1312116 J Gen Virol, 1992
- The primase activity of the intertypic complex was 10 fold lower than that of HSV 1 UL5 HSV 1  **UL8**  HSV 1  **UL52** .  Ref: 9445019 J Virol, 1998
- Herpes simplex virus type 1 encodes a heterotrimeric helicase primase composed of the products of the UL5,  **UL52** , and  **UL8**  genes.  Ref: 9020191 J Biol Chem, 1997
- The herpes simplex virus helicase primase complex, a heterotrimer of the UL5,  **UL8** , and  **UL52**  proteins, displays a single predominant site of primer synthesis on phi X174 virion DNA Tenney, D.  Ref: 7721827 J Biol Chem, 1995
- Herpes simplex virus type 1 HSV 1 encodes a heterotrimeric helicase primase composed of the products of the three DNA replication specific genes UL5,  **UL8** , and  **UL52**  Crute, J.  Ref: 7775476 J Biol Chem, 1995
- We show that the HSV 1 UL5,  **UL8** , UL29, UL30, UL42, and  **UL52**  gene products along with the AAV Rep68 protein are sufficient to initiate replication on duplex DNA containing the AAV origins of replication, resulting in products several hundred nucleotides in length.  Ref: 11581393 J Virol, 2001
- Herpes simplex virus type 1 encodes a heterotrimeric helicase primase complex composed of the products of the UL5,  **UL52** , and  **UL8**  genes.  Ref: 11278618 J Biol Chem, 2001
- The intertypic complex HSV 2 UL5 HSV 1  **UL8**  HSV 1  **UL52**  was purified and biochemically characterized.  Ref: 9445019 J Virol, 1998
- The herpes simplex virus type 1 DNA helicase primase is a heterotrimer encoded by the UL5,  **UL8** , and  **UL52**  genes.  Ref: 8702953 J Biol Chem, 1996
- The UL5,  **UL8** , and  **UL52**  genes of herpes simplex virus type 1 encode a multisubunit assembly that possesses primase, DNA helicase, and DNA dependent nucleoside triphosphatase activities.  Ref: 9013584 J Biol Chem, 1997
- The products of herpes simplex virus type 1 HSV 1 genes UL5,  **UL8**  and  **UL52**  form a complex in virus infected cells that exhibits both DNA helicase and DNA primase activities.  Ref: 7931156 J Gen Virol, 1994
- While the  **UL8**  component of the helicase primase is not required for enzymatic activities by a subassembly of the UL5 and  **UL52**  proteins, only the holoenzyme UL5 8 52 was stimulated by ICP8.  Ref: 9129659 J Gen Virol, 1997
- Since the UL5 and  **UL52**  proteins together form a complex which is enzymatically indistinguishable from a complex formed from all three proteins, a possible role of the  **UL8**  protein may be in facilitating nuclear uptake.  Ref: 1312116 J Gen Virol, 1992
- The results shown in this paper are consistent with the model that the five proteins, ICP8, UL5,  **UL8** , UL9, and  **UL52** , form a scaffold.  Ref: 12634381 J Virol, 2003
- The ordered assembly of the herpes simplex virus HSV type 1 replication apparatus leading to replication compartments likely involves the initial assembly of five viral replication proteins, ICP8, UL9, and the heterotrimeric helicase primase complex UL5  **UL8**   **UL52** , into replication foci.  Ref: 12634381 J Virol, 2003
- An assembly of the UL5 and  **UL52**  subunits retains both enzymic activities, and the  **UL8**  protein has been implicated in modulating these functions, facilitating efficient nuclear uptake of the complex and interacting with other viral DNA replication proteins.  Ref: 9344911 Virology, 1997
- The herpes simplex virus type 1 HSV 1 helicase primase, an essential component of the viral DNA replication machinery, is a trimeric complex of the virus coded UL5,  **UL8** , and  **UL52**  proteins.  Ref: 9344911 Virology, 1997
- Herpes simplex virus type 1 encodes a heterotrimeric helicase primase complex that is composed of the products of the UL5,  **UL52** , and  **UL8**  genes.  Ref: 10075707 J Biol Chem, 1999
- the heterotrimeric helicase primase complex, encoded by UL5,  **UL8** , and  **UL52**  genes, is believed to unwind duplex viral DNA at replication forks.  Ref: 11642367 Prog Nucleic Acid Res Mol Biol, 2001
